# Prevalence and Characterization of Murine Leukemia Virus Contamination in Human Cell Lines

**DOI:** 10.1371/journal.pone.0125622

**Published:** 2015-04-30

**Authors:** Cord C. Uphoff, Sandra Lange, Sabine A. Denkmann, Henk S. P. Garritsen, Hans G. Drexler

**Affiliations:** 1 Department of Human and Animal Cell Lines, Leibniz Institute DSMZ—German Collection of Microorganisms and Cell Cultures, Braunschweig, Germany; 2 Institute for Clinical Transfusion Medicine, Municipal Hospital, Braunschweig, Germany; Harvard Medical School; Massachusetts General Hospital, UNITED STATES

## Abstract

Contaminations of cell cultures with microbiological organisms are well documented and can be managed in cell culture laboratories applying reliable detection, elimination and prevention strategies. However, the presence of viral contaminations in cell cultures is still a matter of debate and cannot be determined with general detection methods. In the present study we screened 577 human cell lines for the presence of murine leukemia viruses (MLV). Nineteen cell lines were found to be contaminated with MLV, including 22RV1 which is contaminated with the xenotropic murine leukemia virus-related virus variant of MLV. Of these, 17 cell lines were shown to produce active retroviruses determined by product enhanced reverse transcriptase PCR assay for reverse transcriptase activity. The contaminated cell lines derive from various solid tumor types as well as from leukemia and lymphoma types. A contamination of primary human cells from healthy volunteers could not be substantiated. Sequence analyses of 17 MLV PCR products and five complete MLV genomes of different infected cell lines revealed at least three groups of related MLV genotypes. The viruses harvested from the supernatants of infected cell cultures were infectious to uninfected cell cultures. In the course of the study we found that contamination of human genomic DNA preparations with murine DNA can lead to false-positive results. Presumably, xenotransplantations of the human tumor cells into immune-deficient mice to determine the tumorigenicity of the cells are mainly responsible for the MLV contaminations. Furthermore, the use of murine feeder layer cells during the establishment of human cell lines and a cross-contamination with MLV from infected cultures might be sources of infection. A screening of cell cultures for MLV contamination is recommended given a contamination rate of 3.3%.

## Introduction

Human and animal cell cultures are highly susceptible to a multitude of contaminations. These comprise cross-contamination with human or animal cells from other cell lines, leading to mixed cell populations or a complete replacement of the original cells by the contaminating cells [1]. Other contaminations are caused by microorganisms like yeast or fungi and bacteria. Special attention should be paid to infections caused by mycoplasmas and mycobacteria among the bacterial contaminations. These organisms are growing very slowly and cannot be detected during routine cultivation of the cells [2].

Although cell lines are commonly used for the production of viruses and for the investigation of virus infections, only sporadic reports address the possible problem of unintended viral contamination of cell cultures. One reason for this dearth of attention may be the generally assumed species- and tissue-specificity of the viruses. A viral contamination is usually predicted to originate from the donor of the cells of a cell culture. Thus, either the donor organism or the cells are screened for potential virus infections, such as Epstein-Barr virus (EBV), hepatitis B virus (HBV), hepatitis C virus (HCV), human immunodeficiency virus (HIV), human T-cell lymphotropic virus (HTLV) or other suspected human pathogenic viruses. The screening for viruses is usually motivated by safety reasons [3]. Animal cells are rarely screened for contaminating viruses because animal viruses are usually not pathogenic to man (with the exception of monkey or bat cell cultures that are known to be reservoirs for human pathogenic viruses).

Indeed, it had been shown that the risk of unknown infection of human continuous cell cultures with the aforementioned viruses is extremely low. Only EBV is significantly prevalent in human B cell lines because this virus is widely distributed among the human population and is also used to immortalize B cells in vitro to generate B lymphoblastoid cell lines. Other human pathogenic viruses can only be found sporadically (e.g. human papilloma viruses, human herpesvirus type 8) and are not further disseminated among other cell cultures [3].

Nevertheless, at least one group of viruses was identified to be able to infect cells from numerous species and various tissues in vitro and to occur in cell cultures: the xeno- and polytropic murine leukemia viruses (X/P-MLV) [4]. The mouse leukemia virus types belong to the gamma-retroviruses and display diverse host range tropisms defined by the expression of different envelope surface proteins interacting with the appropriate receptors of the host cells. One group can only infect mouse and rat cells via the mCAT1 receptor and was designated “ecotropic MLV”. A second group of MLV is unable to infect cells of the originating host species, but can infect cells from other mammals using the XPR1 receptor. These viruses are called “xenotropic MLV”. The third group exhibits the broadest host range and can infect rodent cells as well as cells from other mammalian species binding to XPR1 receptors with different sequence polymorphisms. These viruses are termed “polytropic MLV”. A fourth group of MLV called the “amphotropic MLVs” also infect most mammalian cells, but interact with the SLC20A2 (PiT-2, Glvr-2) receptor, whereas another subgroup interacts with both, SLC20A1 and SLC20A2 (10A1 virus class) [5]. The different groups can be distinguished by interference assays because infected cells are resistant to superinfections with an MLV of the same group, whereas they can be superinfected by viruses which bind to other receptors [6].

In the past decades, several studies were published describing the contamination of cell lines with MLV. Mouse cells usually contain several full-length copies of the different host-range MLVs endogenously in their genome and produce active viruses [7]. Regarding human cells, several individual human cancer cell lines were described to harbor MLV sequences and sometimes to express the viruses (summarized in [8]). Some notoriety gained the detection of xenotropic murine leukemia virus related virus (XMRV). Although correctly detected and characterized in the cell line 22RV1, XMRV was erroneously detected in primary cells from patients with prostate cancer and chronic fatigue syndrome due to contamination of the assay [9].

In the course of screening the cell lines of our biological resource center (BRC) for XMRV we detected MLV contamination in a number of human cancer cell lines. More than 3% of the investigated cell lines were found to harbor MLV sequences and most of them are also positive for reverse transcriptase activity, indicating the production of active viruses. Most of the cell lines were presumably contaminated by xenotransplantation into mice to study the tumorigenicity of the cells. We sequenced a number of the viruses and found a high variability in specific genome regions. We also performed stimulation and cross-contamination tests to study a possible inducibility of expression and the infectiousness of any viruses released during routine cell culture.

## Materials and Methods

### Culture of Cell Lines

The continuous cell lines were provided for accessioning to the BRC by the original or secondary investigators [10]. Cell lines were grown at 37°C in a humidified atmosphere of air containing 5% CO_2_. The basic growth media (Life Technologies, Darmstadt, Germany) were supplemented with 10–20% fetal bovine serum (Sigma Aldrich, Taufkirchen, Germany). For growth factor-dependent cell lines, specific growth factors or conditioned media containing growth factors were added. No antibiotics were added to the cultures. All cell lines were free of mycoplasma and other bacterial, yeast and fungi contaminations as tested by PCR and microbiological growth assays [11]. The authenticity of the cell lines was determined by DNA typing [1]. For the stimulation or induction of the replication competence of XMLV, PCR-positive cell lines were treated for 3–5 days with 10^–7^ M 12-O-tetradecanoylphorbol-13-acetate (TPA) (Sigma Aldrich) and 3 mM Na-butyrate (Sigma Aldrich) or for 3–5 days with 1.25 μg/μl 5´-azacytidine (Sigma Aldrich).

### PCR Assays

PCR was applied for the detection of proviral MLV sequences in the genomes of the human cell lines. Genomic DNA was isolated from PBS-washed cell pellets applying the High Pure PCR Template Preparation kit from Roche (Mannheim, Germany) according to the recommendations of the manufacturer. The DNA of 1 to 5 x 10^6^ viable cells was eluted from the columns with 200 μl dH_2_O. The DNA concentration and purity was determined using a Nanodrop 1000 fluorometer. Approximately 200 to 500 ng of the isolated DNA were used for PCR amplification. The integrity of the DNA was demonstrated by performing short tandem repeat cell line authenticity assays as described elsewhere [1] and by amplification of the human ABL gene as published before [3] (for primer sequences see Table 1).

**Table 1:** Primer Sequences.

Primer name	Assay	Orientation	Sequence (5’– 3’)
F-ABL-A2	Human DNA control	Forward	TGA CTT TGA GCC TCA GGG TCT GAG TGA AGC C
R-ABL-A3	Human DNA control	Reverse	CCA TTT TTG GTT TGG GCT TCA CAC CAT TCC
IAP-F	Mouse DNA	Forward	ATA ATC TGC GCA TGA GCC AAG G
IAP-R	Mouse DNA	Reverse	AGG AAG AAC ACC ACA GAC CAG
PERT-RT2F	PERT	Forward	TCC TGC TCA ACT TCC TGT CGA G
PERT-RT1R	PERT	Reverse	CAC AGG TCA AAC CTC CTA GGA ATG
XMRV out F*	MLV diagnostic PCR	Forward	CCG TGT TCC CAA TAA AGC CT
XMRV out R	MLV diagnostic PCR	Reverse	TGA CAT CCA CAG ACT GGT TG
XMRV in F	MLV diagnostic PCR	Forward	GCA GCC CTG GGA GAC GTC
XMRV in R	MLV diagnostic PCR	Reverse	CGG CGC GGT TTC GGC G
XMLV-650F	sequencing	Forward	ATG GGA CAG ACC GTA ACC AC
XMLV-1200F	sequencing	Forward	CCA GGC ATT CCC ACT CCG CAT
XMLV-1300F	sequencing	Forward	TGA AGA TCC AGG TAA ATT GAC TGC C
XMLV-1700F	sequencing	Forward	GAG ACT CAA GGA GGC CTA TCG
XMLV-2500R	sequencing	Reverse	CAG TCT GGC TTC CTG TGA CAT
XMLV-2700F	sequencing	Forward	TTC GCC AAG CGC CTC TGA TTA
XMLV-3400F	sequencing	Forward	ACG GCA GGC TTC TGT CGC CT
XMLV-3400R	sequencing	Reverse	AGG CGA CAG AAG CCT GCC GT
XMLV-3900F	sequencing	Forward	CCA TGC AGT AGA GGC ACT GGT
XMLV-4077F^#^	sequencing	Forward	GAT TGC CTC GAG ATC TTG GC
XMLV-4096R*	sequencing	Reverse	GCC AAG ATC TCG AGG CAA TC
XMLV-4700F	sequencing	Forward	CTG GGA GCC ACC TAT AAT CAG AT
XMLV-5200F	sequencing	Forward	CAG GTA AGT CAT TCG GTG GCC
XMLV-5900F	sequencing	Forward	CAG CCC TCA CCA GAT CTT CAA TG
XMLV-6800R	sequencing	Reverse	GGG TCA GCT TGT GTT GGG AGG
XMLV-7400R	sequencing	Reverse	AGT GTG GTC CGC GTA GAA AC
XMLV-7450F	sequencing	Forward	GAA CCG GAG AGG ATT AGA TCT GC
XMLV-7900F	sequencing	Forward	AAG AAC CGA TGG TGC CCA CCT
XMLV-8161R^#^	sequencing	Reverse	CGA CTC AGT CTA TCG GAG GA
XPR1-out-F	Receptor PCR	Forward	CAC TGG TGT TAC TAC GCG T
XPR1-out-R	Receptor PCR	Reverse	GCA ACA AAG TTG TAG AGG T

* **Primers used for preparing 5´-half of the MLV genome for sequencing**

^**#**^ **Primers used for preparing 3´-half of the MLV genome for sequencing**

The study included backup blood samples which are drawn from each donor on a regular basis. No additional blood drawing or sampling was necessary for this study. The samples were anonymized before testing and the institutional review board of the Institute for Clinical Transfusion Medicine, Städtisches Klinikum Braunschweig gGmbH, Braunschweig, Germany, approved this procedure. The study was performed in accordance with the Declaration of Helsinki. There is a written informed consent for this study for each of the participants.

#### MLV diagnostic PCR

Specific oligonucleotides spanning the 5´-untranslated region and the beginning of the gag-gene of MLV/XMRV were used to amplify a ca. 657 to 681 bp fragment of MLV. The exact size of the fragment depends on the MLV strain. A second primer pair was used to increase the sensitivity and specificity in a nested PCR for amplification of a PCR product of 175 to 199 bp (for primer sequences see Table 1). Critical for a sensitive and reproducible PCR run are the following conditions for both reactions of the nested PCR: the PCR mixture contained 100 μM of each dNTP, 0.4 μM each of forward and reverse primers, 1 x PCR buffer as recommended by the manufacturer of the Taq polymerase, 5% formamide and 1 U TaKaRa HS Taq polymerase (Lonza, Verviers, Belgium). One μl genomic DNA was added at last. The Taq polymerase was activated by heating the mixture for 2 min at 96°C. The DNA amplification was carried out for 35 cycles with 4 s at 96°C denaturation, 8 s at 60°C annealing, 15 s plus 1 s per cycle at 72°C extension, and 10 min at 72°C as final extension at the end of the PCR. The nested PCR with the inner primers was carried out employing the same parameters, except that 1 μl of the first round PCR mixture was added instead of the genomic DNA and the PCR was run for only 30 cycles. Positive, negative, and water control reactions were included in every PCR run.

#### PCR for MLV genome sequencing

Full length viral genomes from the Entrez Genome Browser online database (www.ncbi.nlm.nih.gov) were imported into Vector NTI sequence analysis program (Life Technologies) and aligned. Conserved regions were chosen to design oligonucleotides for the amplification of DNA fragments longer than 4 kbp. Further oligonucleotides were designed to produce type-specific DNA fragments for sequencing. All primers used for amplifying long PCR fragments and for sequencing are listed in Table 1. The PCR mixture contained 100 μM of each dNTP, 0.4 μM each of forward and reverse primers, 1 x PCR buffer as recommended by the manufacturer of the Taq polymerase, 20% betaine (Sigma Aldrich) and 1 U TaKaRa HS ExTaq polymerase (Lonza). The DNA was added at last. The Taq polymerase was activated by heating the mixture for 2 min at 96°C. The DNA amplification was carried out for 35 cycles with 30 s at 96°C denaturation, 30 s at 60°C annealing, 3 to 4 min plus 2 s per cycle at 72°C extension (depending on the expected PCR product size), and 10 min at 72°C as final extension at the end of the PCR.

#### Mouse-IAP-PCR

PCR for the demonstration of intracisternal A particles (IAP) to detect mouse genomic DNA was performed essentially as described for the diagnostic MLV PCR, except for the exchange of MLV primer by IAP primer and the use of 20% betaine instead of 5% formamide.

#### XPR1 Reverse Transcriptase PCR

Total cellular RNA of the cell lines VERO-B4, NCI-H82, and MEL-JUSO was prepared employing Trizol reagent (Life Technologies) according to the manufacturer’s recommendations. Five micrograms of total RNA were used as a template for first strand cDNA synthesis applying a reverse transcriptase preamplification system kit (DyNAmo cDNA Synthesis Kit; Thermo Scientific, Dreieich, Germany). The reverse transcription was carried out with oligo dT primers according to the recommendations of the manufacturer. Two microliters of the first strand cDNA reaction were then used in a PCR for the detection of XPR1 transcripts which are the cellular receptors for the binding of MLV. The PCR was performed as described above for the diagnostic MLV PCR, but with specific XPR1 primers (Table 1), TaKaRa HS ExTaq polymerase with the respective PCR buffer and without formamid.

### Product Enhanced Reverse Transcription PCR Assay (PERT)

The PERT assay was used to sensitively demonstrate the activity of retroviral reverse transcriptase (RT) in cell culture supernatants. The potentially present viral RT is used to produce a cDNA strand initiated by an artificial primer template hybrid consisting of MS2 bacteriophage RNA and an MS2-specific primer. The cDNA is then amplified with a second MS2-specific primer in a PCR reaction.

#### Preparation of cell culture supernatant

Five ml of conditioned cell culture supernatant were harvested and centrifuged for 30 min at 2000 x g and 4°C to remove cells and cell debris. One hundred μl of the supernatant were mixed with 100 μl of PERT lysis buffer A (50 mM KCl, 25 mM Tris-Cl pH 7.5, 5 mM DTT, 0.25 mM EDTA pH 8.0, 0.025% Triton X-100, 50% glycerin) and 1 μl 10% Triton X-100 [12]. The mixture was frozen at -80°C and thawed at 37°C to release the RT from the viruses.

#### Primer template annealing

One μl of PERT-RT1R primer at 10 mM was mixed with 0.4 μl bacteriophage MS2 RNA at 0.8 μg/μl (Roche Diagnostics, Mannheim, Germany) per reaction, denatured at 95°C for 5 min and subsequently incubated for 30 min at 37°C for annealing of the primer to the template. The primer template mixture was chilled on ice and processed immediately or stored at -20°C.

#### Reverse transcription reaction

Two RT reactions were carried out per sample. Five μl of the lysis reaction were combined with 23.6 μl of 1 x Mg^2+^-RT-buffer and 1.4 μl of primer-template mixture or with 15 μl 2 x Mn^2+^-RT buffer, 1.4 μl of primer-template mixture and 8.6 μl dH_2_O. The 1 x Mg^2+^-RT-buffer consisted of 56 mM KCl, 56 mM Tris-Cl pH 8.3, 9.2 mM MgCl_2_, 11.2 mM DTT, 0.13 μg/μl BSA, and 1 mM each of dNTPs [12]. The 2 x Mn^2+^-RT buffer consisted of 60 mM KCl, 20 mM HEPES pH 7.2, 6 mM MnCl_2_, 4 mM spermine tetrahydrochloride, 1% triton X-100, 0.4 mM EDTA, 6 mM β-mercaptoethanol, 1 mg/ml BSA and 1 mM each of dNTPs. Then 0.1 U/μl Ribolock RNase inhibitor was added to each of the RT buffers immediately before setting up the RT reaction. The samples were incubated at 37° for 2 h. Two μl of each reaction were either directly subjected to a PCR amplification or after ethanol precipitation and redilution in 5 μl dH_2_O. The PCR was carried out with 100 μM of each dNTP, 0.4 μM each of PERT-RT1R and PERT-RT2F primer, 1 x PCR buffer as recommended by the manufacturer of the Taq polymerase, and 1 U TaKaRa HS ExTaq polymerase. The DNA was added at last. The Taq polymerase was activated by heating the mixture for 2 min at 96°C. The DNA amplification was carried out for 35 cycles with 10 s at 94°C denaturation, 20 s at 55°C annealing, 30 s plus 1 s per cycle at 72°C extension, and 7 min at 72°C as final extension at the end of the PCR run. The PCR products were then analyzed on a 1.3% agarose gel.

### Sequencing of PCR products

Fifty microliter PCR reactions were prepared using genomic DNA and the appropriate primer combination (Table 2) to amplify two PCR products of more than 4 kbp each covering almost the complete genome of MLV. After the PCR run, the DNA in the reaction mixture was size-separated in a 1% agarose gel. The band of the expected size was cut out and the DNA was extracted from the gel employing the QIAquick gel extraction kit (Qiagen, Hilden, Germany) according to the manufacturer’s recommendations. The DNA was eluted with 50 μl dH_2_O and subsequently ethanol precipitated. The DNA was then resuspended in 15 μl dH_2_O. The DNA concentration was determined with a Nanodrop 1000 instrument. Approximately 200 ng of the purified DNA were combined with 15 pmol of the respective sequencing primer in a volume of 15 μl. The sequencing was performed by Eurofins Genomics (Ebersberg, Germany). Sequence analysis was performed with the free software applications Vector NTI (Life Technologies), MEGA6 [13], and "The European Molecular Biology Open Software Suite" (EMBOSS)(http://emboss.bioinformatics.nl).

**Table 2:** MLV-positive cell lines tested by nested PCR and PERT-PCR assays.

Cell line	Cell type	MLV-PCR		control PCR		PERT-PCR	
		1^st^ rd	2^nd^ rd	ABL	IAP	Mg^2+^	Mn^2+^
22RV1	prostate cancer	+	+	+	-	+	+
AC-1M46	choriocarcinoma-trophoblast hybrid	-	+	+	-	(+)	+
BT-B	cervix carcinoma (subclone of HELA)	+	+	+	-	-	+
CML-T1^1^	T-cell leukemia	- / -	+ / -	+ / +	+ / -	-	-
COLO-677	small cell lung carcinoma	+	+	+	-	(+)	+
DEL	malignant histiocytosis	+	+	+	-	-	+
EVSA-T	breast carcinoma	+	+	+	-	+	+
HN^1^	oral squamous carcinoma	- / -	+ / -	+ / +	+ / -	n.d.	n.d.
KARPAS-1106P	B-cell non-Hodgkin lymphoma	+	+	+	-	-	-
KELLY	neuroblastoma	-	+	+	-	+	+
KYSE-70	esophageal carcinoma	+	+	+	-	+	+
LAMA-87	chronic myeloid leukema	+	+	+	-	+	+
LCL-HO	B-lymphoblastoid cells	(+)	+	+	-	+	+
LXF-289	lung adenocarcinoma	+	+	+	-	+	+
M07e^1^	acute megakaryoblastic leukemia	+ / -	+ / -	+ / +	+ / -	n.d.	n.d.
ML-1	follicular thyroid carcinoma	-	+	+	-	+	+
NAMALWA.CSN/70	Burkitt lymphoma (subclone)	+	+	+	-	+	+
NAMALWA.KN2	Burkitt lymphoma (subclone)	+	+	+	-	+	+
NCEB-1	mantle cell lymphoma	+	+	+	+*	-	+
SCLC-21H	small lung cell carcinoma	+	+	+	-	+	+
SK-GT-2	gastric fundus carcinoma	+	+	+	-	+	+
SK-MEL-1	melanoma	-	+	+	-	-	-
SPI-801^1^	chronic myeloid leukemia (K-562)	(+) / -	+ / -	+ / +	+ / -	-	-

* The NCEB-1 cell line is known to harbor several mouse chromosomes in the nuclei of the human cells;-, negative; (+), weakly positive; +, positive; ^1^human genomic DNA samples contaminated with mouse derived DNA (new DNA preparations revealed MLV and IAP negative and ABL positive results). n.d. not determined.

## Results

### Detection of MLV in human cell lines and blood samples

In the course of screening prostate cancer and other suspected cell lines for contaminations with XMRV, we established a nested PCR assay with primers amplifying a region upstream of the *gag* start codon as published previously [14]. DNA of the prostate carcinoma cell line 22RV1 was used as positive control. Optimizing the PCR parameters showed that TaKaRa HS Taq polymerase, the respective PCR buffer including 1.5 mM MgCl_2_, and 5% formamid as PCR additive provides highly sensitive reaction conditions. Furthermore, a BLAST search comparing the primer sequences with sequences from the public databases showed for the outer primer for more than 220 entries and for the inner primer for more than 130 entries complete concordance with murine leukemia viruses, particularly with XMLV and XMRV as well as with mouse chromosomal sequences attributed to endogenous MLV.

**Fig 1:**
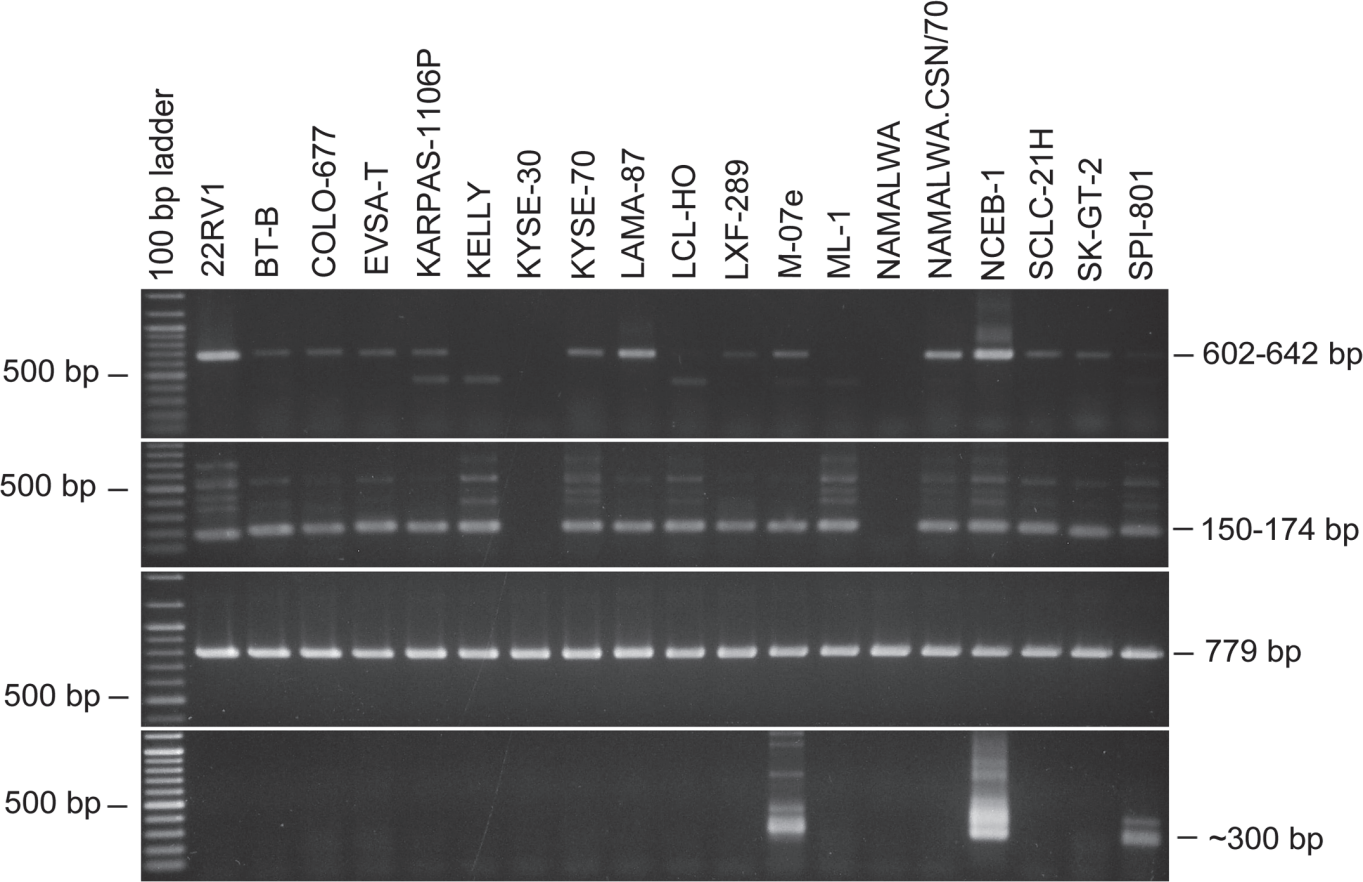
PCR-Assays for the sensitive and reliable detection of MLV sequences in cell line DNA. The upper two panels show the ethidium bromide stained gels of the MLV-specific PCR assays performed with the outer (first panel) and inner primers (second panel). The sizes of the MLV-specific bands are 604–642 bp and 150–174 bp, respectively, depending on the MLV genotype. Some cell lines produce an unspecific band with the outer primers (also seen in some MLV-negative cell lines). The inner primers usually also produce several unspecific bands seen only in MLV-positive cell lines. The cell lines KYSE-30 and NAMALWA are MLV-negative in the assay, whereas the cell lines from the same series or subclones are MLV-positive, respectively. The third panel demonstrates the integrity of the DNAs used for the analyses by amplification of the human ABL gene. The lower panel shows contamination of the genomic DNA with mouse DNA by amplification of mouse specific IAP coding sequences. The PCR produces a double band of approximately 300 bp. The cell line NCEB-1 harbors several mouse-derived chromosomes.

Although none of the other prostate carcinoma cell lines tested (n = 5) were found to be positive for XMRV/MLV, the first PCR assays surprisingly uncovered a few cell lines that were positive for MLV sequences (KARPAS-1106P, LAMA-87). This finding encouraged us to screen all human cell lines of the collection with the nested PCR assay for MLV sequences. Finally, 23 out of 577 human cell lines were positive for MLV-sequences applying nested PCR (S1 Table). However, as shown later, only 19 of these cell lines (3.3% of all tested cell lines) turned out to be contaminated with MLV, whereas the DNA samples of the remaining four cell lines were found to be contaminated with mouse genomic DNA. New DNA preparations of these cell lines were then negative for MLV proviruses. Fourteen of the truly positive cell lines (2.4% of all tested cell lines) were already clearly positive after the first round of PCR, whereas one cell line was weakly positive and four became positive after the second round of PCR using the inner primer pair. One of the false-positive cell lines was positive after the first round of PCR, one was weakly positive and two cell lines were positive after the second round of PCR. Table 2 lists all cell lines positive for MLV proviruses detected by nested PCR. An ethidium bromide-stained gel with MLV-positive and-negative cell lines after the first and the second round of PCR is shown in Fig 1.

The MLV-positive cell lines were not restricted to specific types of cancer, but included cell lines of different leukemia and lymphoma as well as several kinds of carcinoma types. Furthermore, we found closely related cell lines (e.g. subclones, sublines, or sister cell lines) of which one or more were MLV-positive, whereas others were MLV-negative. For example, BT-B, a subclone of the HELA cell line was MLV-positive, whereas HELA itself and other HELA subclones were all MLV-negative. Similarly, only two out of five NAMALWA subclones were MLV-positive.

We also investigated four samples of the cell line A2780 obtained from four different laboratories (not listed in Table 2). Three of them were MLV-negative, whereas one cell culture was MLV-positive. The positive cell line was known to be passaged through a mouse as xenotransplantation. The cell line A2780 had been described previously as MLV-negative [15]. The EKVX cell line (also not listed in Table 2 as it is not part of the cell lines bank) which had been reported to be XMLV-positive [15, 16] was confirmed to be XMLV-positive.

As a first step we also examined a cohort of 30 individuals. We tested genomic DNA isolated from blood samples of 30 healthy volunteers for the presence of MLV sequences by the nested PCR assay. All samples were invariably negative for MLV proviruses. The integrity of the DNAs was verified by amplifying a fragment of the human ABL gene. It is obvious that a larger sampling is required in order to exclude definitively any infection by MLV of blood of apparently healthy subjects as well as in diseased patients.

#### Sequencing of the MLV PCR products

**Fig 2:**
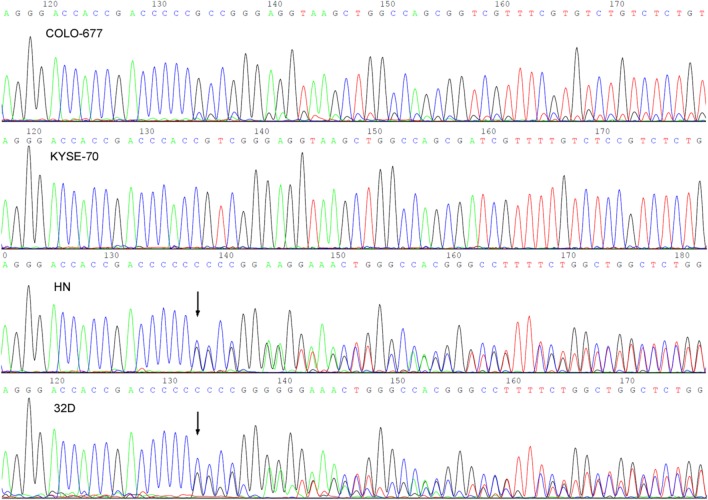
Direct DNA Sanger sequencing chromatograms showing sequence polymorphisms in the human cell lines COLO-677, KYSE-70, HN, and the mouse- derived cell line 32D. COLO-677 and KYSE-70 contain single sequences of MLV contaminants, whereas HN and 32D show identical chromatograms, indicating that the DNA of the human cell line was contaminated with mouse DNA. A single nucleotide deletion (arrows) results in a sequence shift leading to an overlap of two MLV sequences present in the mouse genomic DNA.

The screening of a multitude of DNA samples particularly by nested PCR always bears the risk of generating false-positive results due to contamination of PCR reagents by DNA carry-over. Although maximal care was taken to avoid DNA carry-over, we nevertheless validated the PCR screening results by sequencing the PCR products of those cell lines which produced sufficient PCR product in the first round of amplification. Nineteen PCR products were sequenced in one or both directions applying the outer forward and reverse primers (XMRV-out-F and XMRV-out-R). Seventeen of the sequences revealed unequivocal and complete reads as shown in part in the first two panels of Fig 2. However, the two remaining reads were ambiguous with regard to the sequence approximately 160 bp downstream of the forward primer and 130 bp upstream of the reverse primer. On visual reinspection of the sequence plots we noticed that the equivocal sequence parts originated from overlays of two related sequences. As shown in Fig 2 (third panel), a single nucleotide deletion in a stretch of six cytosine residues causes a sequence shift in one of the sequences approximately 160 bp downstream of the forward primer. Subtracting one MLV sequence from the overlay leaves another closely related MLV sequence behind. Thus, the PCR product consisted of at least two highly related PCR fragments at similar amounts. Furthermore, regarding the above mentioned nucleotide deletion, both sequence types can be identified as single infections in the unequivocal sequence plots. Artifacts of the Taq polymerase or the sequencing reaction are unlikely because analogous results occurred with the reverse primers, the corresponding cytosine stretches were correctly analyzed in the other samples without errors, sequence polymorphisms were identified in other cell lines, and validation experiments produced the same results.

Interestingly, sequence analysis of the PCR products of mouse cell lines (7-TD-1, 32D, BA-F3, F9, GG, N18TG2, and NIH-3T3) invariably produced the same sequence mixture as seen with the ambiguous sequences of the human cell lines (Fig 2D), which initially indicated a double infection with different MLV types. As shown below, the double infections coincide with mouse DNA contaminations of the genomic DNA preparations. Most probably, the two amplification products originate from the mouse genomic DNA and represent endogenous MLV sequences of the mouse.

**Fig 3:**
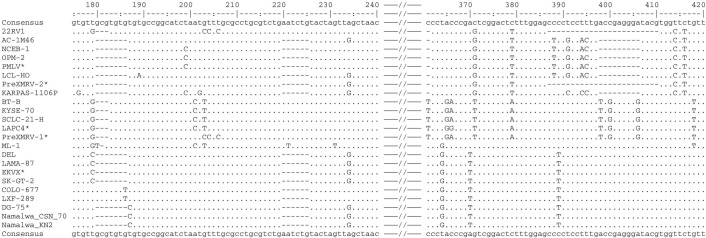
Nucleotide sequence alignment of MLV infected cell lines. The multiple sequence alignment was performed using Vector NTI. The multiple sequence file was then imported to showalign of the EMBOSS program collection. The numbers determine the position from the forward primer. Missing nucleotides are represented by a dash and nucleotides different to the consensus sequence are represented by the respective character. * Cell line sequences originating from the NCBI database.

**Fig 4:**
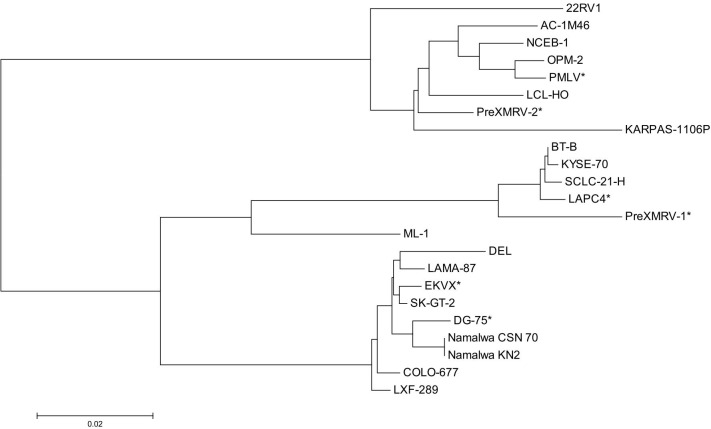
Neighbor joining tree of the MLV sequences of the first round PCR products. The chart demonstrates the relationship of the MLV sequences generated with the outer primer pair of the detection PCR reaction. The alignment and the tree construction were performed with the MEGA6 software. Three major groups of MLV subtypes can be identified. The scale bar represents the number of nucleotide substitutions per site. * Sequences obtained from the NCBI sequence database.

An alignment of the unambiguous sequences displayed a number of polymorphisms among the sequences. Striking are some deletions or insertions of some nucleotides found singly or repeatedly in different cell lines: (a) insertions of three, six or seven nucleotide-long stretches can be detected approximately 190 bp downstream from the forward primer in nine cell lines; (b) a six nucleotide-long insertion at approximately position 220 is seen in four cell lines, including 22RV1; (c) at the alignment position of ca. 400 three groups of sequence types can be identified: beside the complete sequence found in 11 cell lines, a 9 bp deletion displayed in five cell lines, and a 24 bp deletion only in the XMRV-positive cell line 22RV1 as already described elsewhere [14] (which is identical to the PreXMRV sequence). These major sequence differences are shown in Fig 3. Constructing a neighbour joining tree from the alignment of the unambiguous sequences applying the MEGAL6 software revealed at least three genotype clusters of MLV types as shown in Fig 4.

A blast search of each of the different sequences in the NCBI public database revealed complete concordance with a number of exogenous and endogenous MLV sequences and with undefined sequences of different mouse chromosomes. The chromosomal sequences presumably represent endogenous murine retroviruses. The results indicate that at least several MLV types are involved in the contamination of human cell lines. Additionally, the sequence diversity confirms the credibility of the PCR assay regarding false-positive results due to DNA carry-over to PCR reagents. Six sequences from the NCBI database were aligned together with the own sequencing results and are distributed across all three MLV groups (Fig 4).

**Fig 5:**
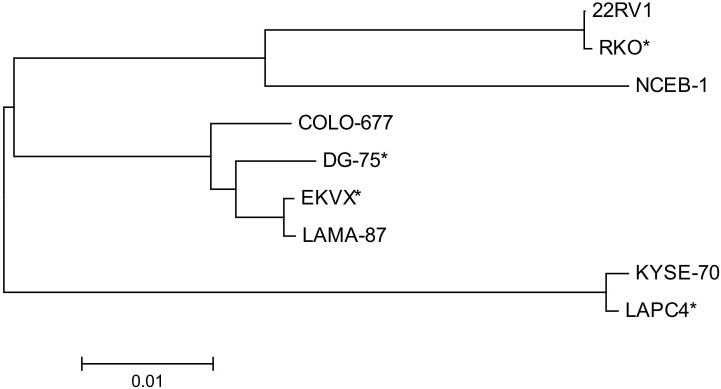
Neighbor joining tree of full genomic MLV sequences. The chart demonstrates the relationship of the complete MLV genomes. The alignment and the tree construction were performed with the MEGA6 software. The investigated sequences produce the same major groups of MLV subtypes as found for the partial sequences. The scale bar represents the number of nucleotide substitutions per site. * Sequences obtained from the NCBI sequence database.

We also performed complete genome sequencing of the five MLV clones from the cell lines 22RV1, COLO-677, KYSE-72, LAMA-87, and NCEB-1 applying primer walking. The primers used for sequencing are listed in Table 1. Per genome sequencing, we first generated PCR products of approximately 4 kbp each applying the primer pairs XMRV-out-F/XMLV-4096R and XMLV-4077F/XMLV-8161R. The PCR products cover the 5´- and the 3´-half of the MLV genome, respectively. The purified PCR products were then used for Sanger sequencing employing different forward or reverse primers to cover the whole MLV genome. Some regions were sequenced in both directions, whereas some regions were sequenced in one direction only. However, the readable regions of the sequences spanned by forward and reverse primers were completely identical. The alignment confirmed the previous finding of different MLV types. They can be allocated to three subgroups of MLV. As already described, the MLV genome consists of conserved sequences spanning the *gag/pro/pol* polyprotein coding region and the *env* region, whereas the sequences of the 5´- and 3´-terminal regions display a higher degree of nucleotide variation [17]. This finding was confirmed in the present study. An over-all alignment revealed a similar evolutionary relationship as the alignment of the 5´-terminal region (Fig 5). As already described for the 5´-untranslated region, the 3´-terminal region also displays several deletions or insertions (sequence duplications), which seem to be characteristic for some MLV clones.

The identity and similarity of the more distantly related viruses of NCEB-1 and LXF-1 average 90%, whereas the closely related viruses of LAMA-87 and DEL average 99% regarding the PCR product of the outer primer pair. Similar identities can be found regarding the complete sequence: 90% for the viruses of the cell lines of NCEB-1 and KYSE-70 and 98% between LAMA-87 and COLO-677 as determined with the program “Matcher” from the EMBOSS sequence analysis software (Figs 4 and 5).

### Release of active viruses by MLV contaminated cell lines

**Fig 6:**
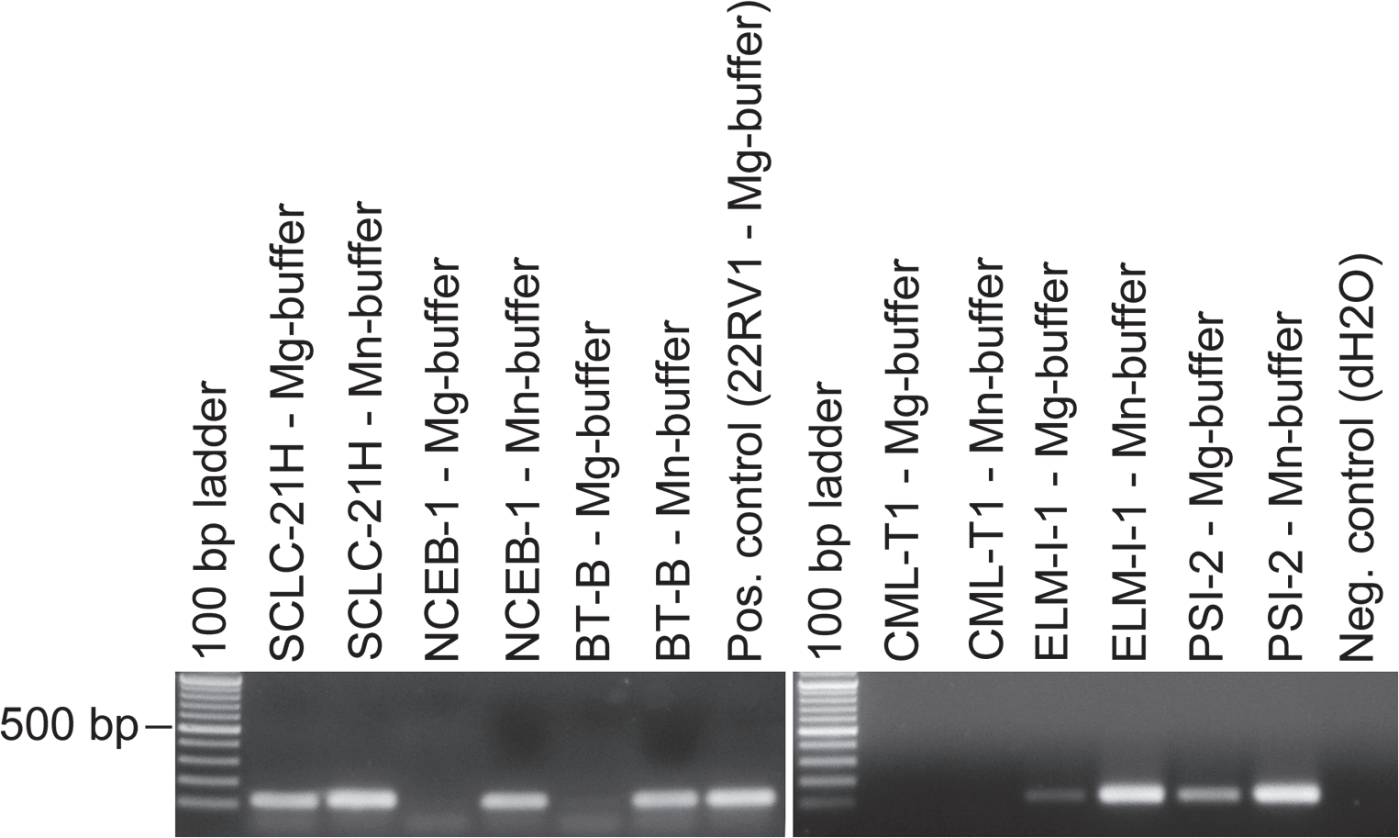
Detection of RT activity in human and mouse (ELM-I-1 and PSI-2) cell culture supernatants by PERT assay. The assay was carried out applying two different buffers containing MgCl_2_ or MnCl_2_, respectively. The Mn^2+^-buffer was more sensitive in most cases, indicating that the contaminating viruses presumably belong to the Mn^2+^-dependent C-type retroviruses. The CML-T1 cell line was MLV-PCR positive due to contamination of the genomic DNA with mouse derived DNA. The size of the MS2 PCR product is 112 bp.

To investigate the release of mature MLV by provirus-positive human cell lines, we performed a product-enhanced reverse transcriptase (PERT) PCR assay as described previously [12]. The method was modified regarding the reaction buffers. The RTs of several gamma-retroviruses (C-type retroviruses) are described to be Mn^2+^-dependent. In addition to the MgCl_2_-buffer we applied a second RT reaction buffer containing MnCl_2_ instead of MgCl_2_ according to the protocol of Malmsten et al. [18]. In contrast to the buffer used by Malmsten et al., optimization of the buffer revealed a higher sensitivity applying 4 mM polyamine concentration instead of 24 mM. An MS2-specific primer was hybridized to RNA of the bacteriophage MS2 and added to the lysed viruses potentially present in the cell culture supernatant and the respective RT reaction buffer for the generation of cDNA. The MS2-specific cDNA is subsequently amplified by PCR with an additional MS2-specific reverse primer. Applying the two RT reaction buffers in parallel reactions, most RT-positive samples produced PCR products with both buffers. However, the PCR product of the Mn^2+^-buffer was usually stronger than the product of the Mg^2+^-buffer reaction. For six samples the RT-reaction was completely Mn^2+^-specific (Fig 6). As shown in Table 2, 17 of the 23 MLV provirus positive cell lines were also positive for reverse transcriptase activity. Two cell lines were identical to those exhibiting overlying MLV sequences (HN and SPI-801). This result supports the assumption that the PCR products originate from mouse DNA contaminations of the genomic DNA as confirmed by mouse IAP PCR (see below). One of the initially mouse IAP PCR positive but after repeated DNA extraction IAP negative cell lines was negative for RT-activity (CML-T1), whereas two cell lines were not further analyzed by PERT-PCR (HN and M07e). They were regarded as initially false positive due to accidental mouse DNA contamination of the DNA preparations. In two cell lines positive for MLV proviruses no RT activity could be demonstrated applying the PERT assay (KARPAS-1106P and SK-MEL-1).

The enzymes of the MLV retroviruses are initially packaged during the replication process of the retrovirus as inactive proteins linked together in the Gag-Pol polyprotein consisting of Gag, the structural protein of the virus capsid, and Pol consisting of the protease, RT, and the integrase. During the following maturation process of the already released complete viruses the proenzyme is proteolytically cleaved to produce the various active enzymes that are necessary for the infection of cells [5]. Thus, the reverse transcriptase activity in the cell culture supernatant is an indicator for the presence of maturated active retroviruses. As all RT-positive cell cultures were detected by the Mn^2+^-buffer, a restriction to this RT reaction buffer might be possible without decreasing the sensitivity and specificity of the assay. The intensities of the PERT-PCR signals are diverse and sometimes weak, indicating variable expression of active viruses by the cell cultures.

### Stimulation experiments for the induction of MLV expression

The weakly PERT-positive cell line SK-GT-2 was treated with the chemical induction reagents 5-azacytidine, and a combination of 12-O-tetradecanoylphorbol-13-acetate (TPA) and sodium butyrate. Although the reagents induced a differentiation of the cells as determined by morphological changes and cessation of cell proliferation, no stimulation of MLV expression was detected employing the PERT assay. According to these results, the activation of infectious viruses during conventional cell cultivation is unlikely.

### Infection of MLV-negative cell cultures with MLV from other cell lines

**Fig 7:**
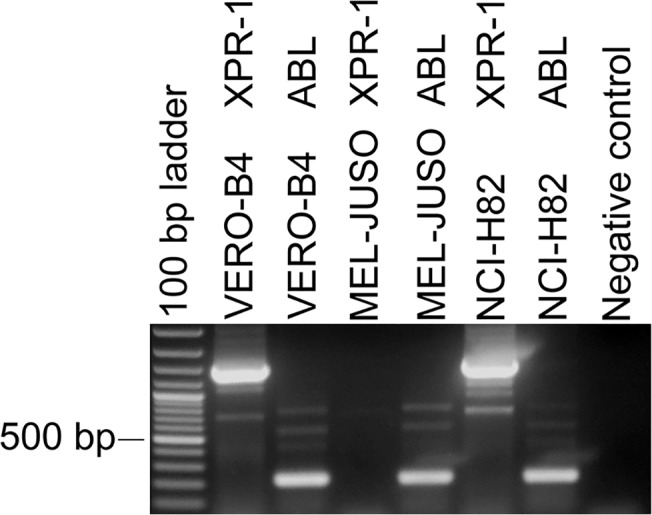
Detection of XPR-1 expression in the monkey cell line VERO-B4 and the human cell lines MEL-JUSO and NCI-H82 by RT-PCR. VERO-B4 and NCI-H82 express XPR-1 mRNA in significant amounts (1455 bp), whereas MEL-JUSO lacks the expression of XPR-1 transcripts. The demonstration of the ubiquitously expressed ABL mRNA was used to show the integrity of the mRNA and the cDNA (216 bp).

To investigate a possible cross-contamination of MLV from one cell culture to another, we performed infection experiments of MLV-negative cell cultures with cell culture supernatants of MLV-positive cell lines. The cell lines 22RV1, COLO-677, DEL, EVSA-T, KELLY, KYSE-70, LCL-HO, NAMALWA.CSN/70, NCEB-1, and SCLC-21H were all shown by PERT PCR assay to release active MLV into the cell culture supernatant. Cell free culture supernatants were used as MLV source to infect the MLV-negative cell lines MEL-JUSO, NCI-H82, and VERO-B4, of which the latter two were previously shown to be sensitive for MLV infections: the VERO-B4 cell line was used for infection studies [16], whereas NCI-H82 was shown to exist as MLV-positive as well as MLV-negative cell culture [19]. Due to the fact that the NCI-H82 clone of the cell bank was retrovirus-negative, we used this human cell line for the infection studies. On the other hand, we were able to demonstrate by RT-PCR that the cell line MEL-JUSO is one of the rare cell lines deficient for XPR-1 mRNA expression (Fig 7). XPR-1 is reportedly the receptor protein for XMLV [4]. The MLV-negative cell cultures were infected with different amounts of viruses: (a) 2 ml of supernatant were filtered through a 0.2 μm filter and directly added to the MLV-negative cell culture; (b) 8 ml of virus-containing cell culture supernatant was initially filtered with a 0.2 μm filter and subsequently ultracentrifuged to concentrate the viruses; the pellet was resuspended in 800 μl PBS and added to the MLV-negative cell cultures. The infected cells were then cultivated for up to six weeks.

**Fig 8:**
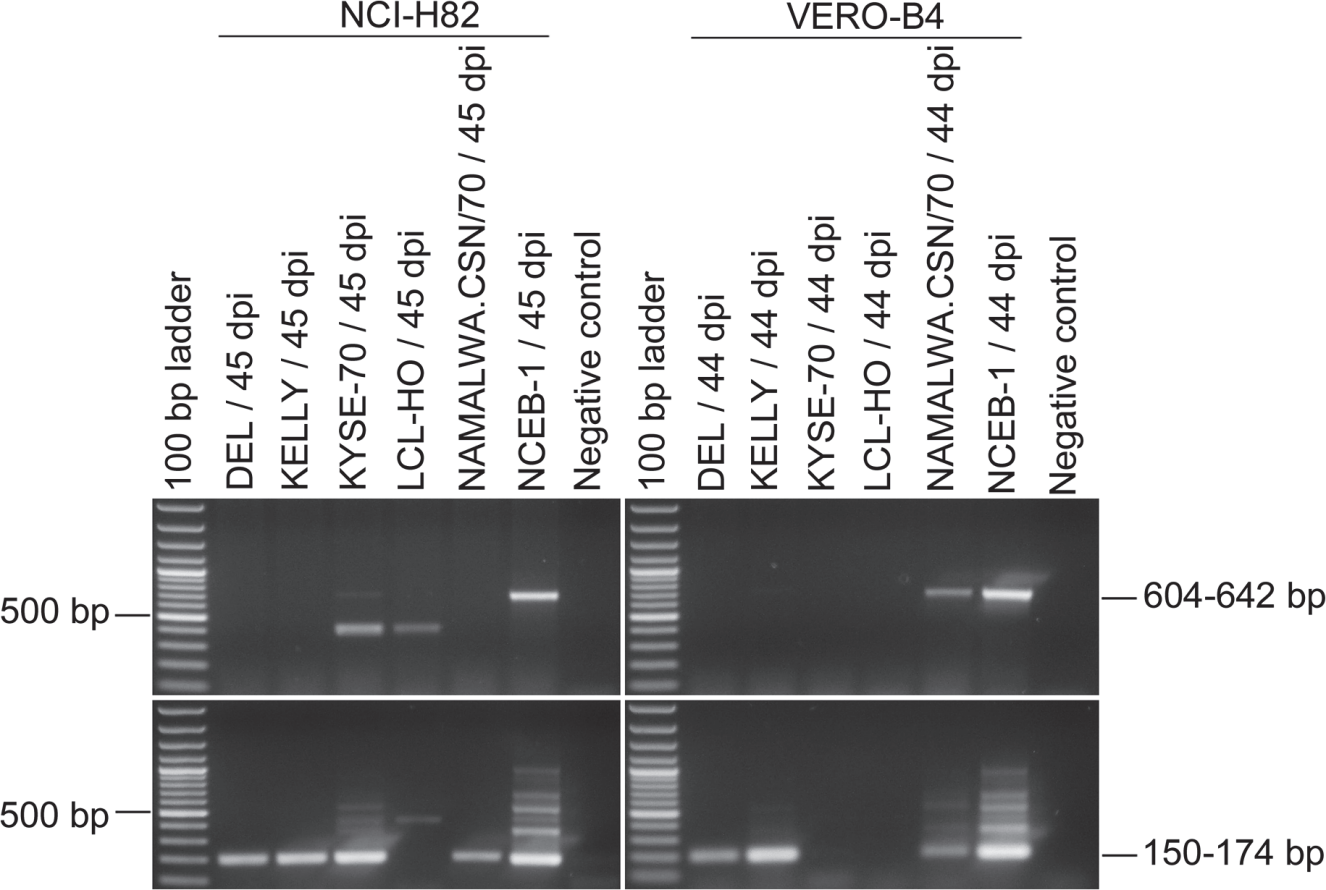
PCR-Assays for the detection of MLV sequences in cell lines infected with MLV from contaminated cell lines. The cell lines NCI-H82 and VERO-B4 were infected with cell culture supernatant of the cell lines DEL, KELLY, KYSE-70, LCL-HO, NAMALWA.CSN/70, or NCEB-1. DNA of the infected cell lines was prepared after 44 or 45 days (dpi, days post infection). NCI-H82 and VERO-B4 were previously shown to be MLV-negative. Only the 604–642 bp bands and the 150–174 bp bands are specific for MLV contaminations determined by the outer and inner primers, respectively.

**Fig 9:**
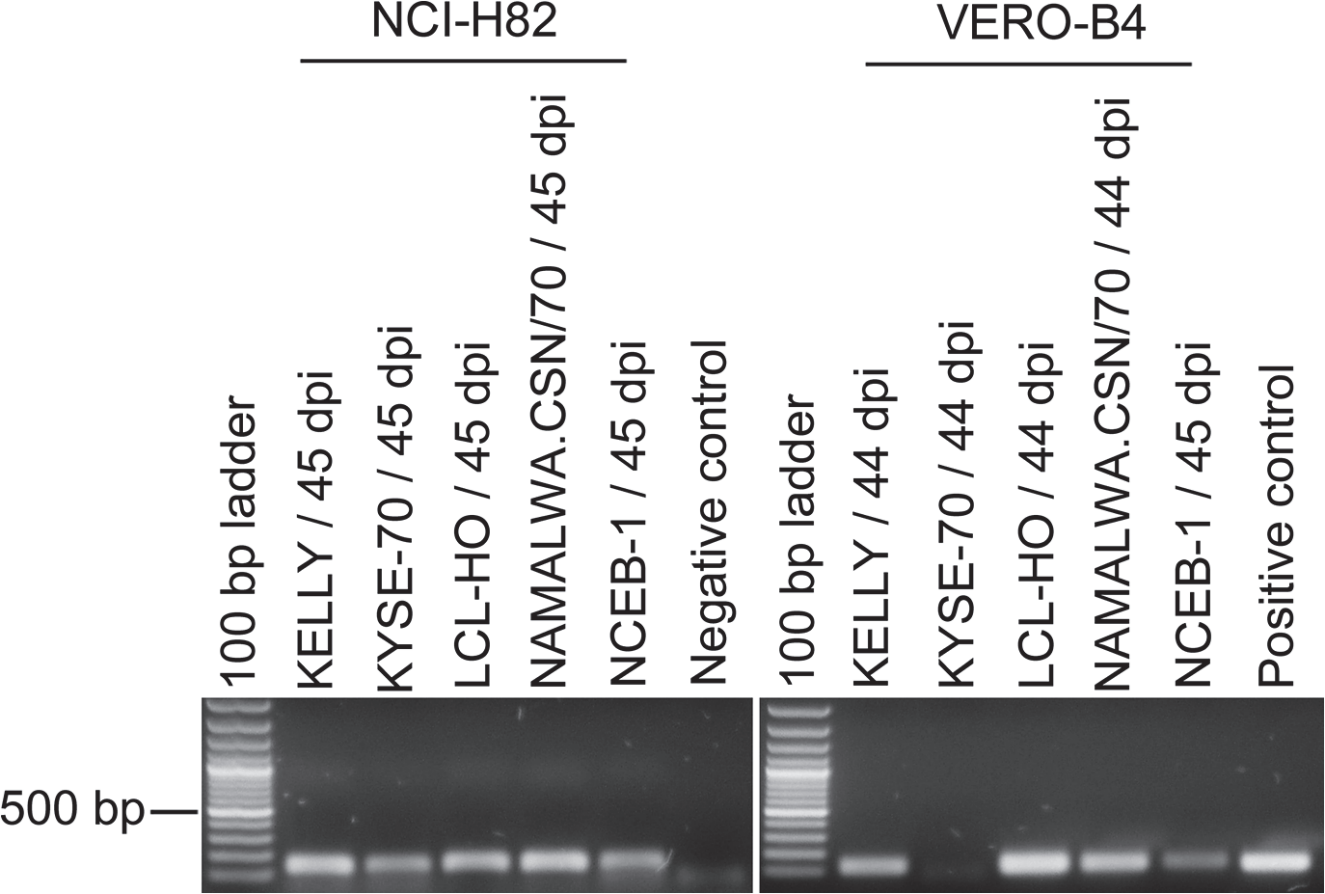
Detection of RT activity in cell lines infected with MLV of contaminated cell lines by PERT assay. Cell culture supernatants of the infected cell lines NCI-H82 and VERO-B4 were harvested after 45 and 44 days after infection, respectively. MS2 phage RNA was reverse transcribed with an MS2-specific primer and the MLV derived RT of the samples in the presence of MnCl_2_. The cDNA was then amplified with a second MS2-specific primer applying PCR. The signals of the cell lines infected with LCL-HO supernatant may potentially be ascribed to the activity of SMRV derived RT. dpi: days post infection.

As shown in Table 3, all infection experiments resulted in an integration of MLV into the eukaryotic genome except for LCL-HO. The extent of infections was highly variable; two out of five exposed NCI-H82 cell cultures showed a PCR signal already after the first round of PCR, whereas the others became positive after the second PCR run (Fig 8). Similarly, only four out of eight infected VERO-B4 cell lines were positive after the PCR assay with the outer primers. Proviruses of only one cell line (LCL-HO) were not detectable at all by PCR in the infected cell lines NCI-H82 and VERO-B4. However, active viruses could be detected in both cell lines for up to 44 or 45 days post infection, respectively (Fig 9). Although we have no absolute proof for a complete washing-out of the inoculated viruses, we consider it unlikely that viruses remain after such a long culture period and many passages. As demonstrated before, the LCL-HO cell line is also infected with squirrel monkey retroviruses [3]. Hence, these viruses might have been transmitted to the cell line and render the PERT-PCR assay positive and the MLV PCR negative. We have no indication from the screening of the cell lines that the PERT-PCR assay is more sensitive than the nested PCR assay.

**Table 3:** Infection experiments of MLV-negative cell lines with MLV from infected cell lines.

Infected Cell Line	Virus Source Cell Line	Infection Status			
		MLV-PCR		PERT-PCR	
		out	in	Mg^2+^	Mn^2+^
VERO-B4	22RV1	+	+	+	+
	DEL	-	+	-	-
	EVSA-T	+	+	+	+
	KELLY	-	+	+	+
	KYSE-70	-	+	-	-
	LCL-HO	-	-	+	+
	NAMALWA.CSN/70	+	+	+	+
	NCEB-1	+	+	+	+
	SCLC-21H	-	+	(+)	(+)
NCI-H82	DEL	-	+	n.d.	n.d.
	KELLY	-	+	+	+
	KYSE-70	+	+	+	+
	LCL-HO	-	-	+	+
	NAMALWA.CSN/70	-	+	+	+
	NCEB-1	+	+	+	+
MEL-JUSO (XPR-1 negative)	22RV1	+	+	+	+
	COLO-677	-	-	-	-

On the other hand, we observed proviruses in two inoculated cell cultures, but without any virus production (VERO-B4/ DEL MLV, VERO-B4/ KYSE-70 MLV) and a third culture with an extremely low virus expression which was hardly detectable with the Mg^2+^ and Mn^2+^-reaction buffer (NCI-H82/SCLC-21H MLV).

MEL-JUSO was infected with cell culture supernatant of the cell lines 22RV1 and COLO-677. Although the cell line does not express XPR-1 surface protein, XMRV of the cell line 22RV1 was able to infect the cells and PERT-PCR demonstrated the presence of active viruses in the virus cross-contaminated cell culture. This result was unexpected, because XMRV was assigned to the xenotropic MLV and should therefore use the XPR-1 membrane protein of the eukaryotic cells for internalization of the viruses. Hence, the virus seems to be able to use an alternative receptor or it does not use XPR-1 at all and has to be assigned to another MLV type. Infection studies of the MEL-JUSO cell line with MLV of COLO-677 revealed no sign of infection, neither integration into the genome of the host cells nor release of active viruses into the cell culture supernatant.

The results indicate that MLV infections can be spread by cross-contamination from an infected culture to a non-infected culture.

### Detection of mouse IAP in human cell lines

Although all human genomic DNAs were previously analyzed for authenticity and cell line cross-contamination by short tandem repeat (STR) analysis [1], we additionally performed a PCR assay for the detection of mouse intracisternal A particles (mouse IAP PCR) to detect with a highly sensitive approach any contamination of human cell cultures with mouse cells or human DNA preparations with mouse DNA. Approximately 1000 IAP elements can be found per haploid mouse genome [20].

Five DNA preparations of human cell lines were found to be positive for both, IAP and MLV. The NCEB-1 cells represent human-mouse hybrid cells and were described to stably carry 5–8 mouse chromosomes in their nuclei. The origin of these chromosomes is still a matter of debate, but it is suspected that they were introduced into the cells by co-culture with mouse feeder layer cells [21, 22]. In contrast to the other IAP-positive cell lines, NCEB-1 sequencing revealed not the typical overlay of at least two MLV sequences as described before (Fig 2). Hence, the NCEB-1 cell line is a truly MLV positive cell line in spite of the detection of IAP sequences in the DNA preparations.

Controlling the DNA preparations, it turned out that the double-positive DNAs had been prepared in parallel with mouse DNAs. Presumably, they had been contaminated with small amounts of the mouse genomic DNAs. Newly prepared DNA was then negative in both PCR assays. In conclusion, identifying MLV sequences in the human genome does not necessarily indicate a virus contamination of the cells. It is also possible that the DNA is contaminated with traces of mouse cell line DNA. Mouse IAP PCR or sequencing of the PCR products can verify the purity of the DNAs.

The contamination of human DNA with traces of murine DNA is concordant with results of a study performed by Zhang et al. [19] who found that three out of 26 DNA preparations were contaminated with murine genomic DNA and with observations of Oakes et al. who described human DNA samples as being contaminated with mouse genomic DNA [23]. Zheng et al. [24] found mouse DNA contamination in commercial human DNA preparations and RT-PCR reagents. Hence, analysis of MLV genomic DNA in human cell lines should always be accompanied by assays for the detection of murine DNA (and perhaps vice versa). The results also indicate that highly sensitive methods are necessary to detect the mouse DNA contaminations, when human and mouse cell lines are concurrently handled in a laboratory. Methods like STR profiling are not sensitive enough to detect those low level contaminations.

## Discussion

Over the last decades several reports have been published on the detection of retroviruses from different mammalian species in human cell lines, indicating contaminations with feline retroviruses [25], murine gamma-retroviruses (reviewed in [8]), and squirrel monkey retroviruses [3]. Most of the reports on the occurrence of MLV in human cell lines describe single infected cell lines [16, 26–28]. However, a few reports deal with the selective detection of MLV in multiple cell lines. Deichmann et al. [29] investigated eight human melanoma cell lines and found four to be MLV-positive by PERT-PCR assay. Takeuchi et al. [30] investigated 16 human cell lines commonly used in human immunodeficiency virus research and detected three MLV-positive cell lines. Zhang et al. investigated a total of 151 different cell cultures including 23 xenograft cultures, 78 non-xenograft cultures grown in xenograft culture-containing facilities, and 50 cell cultures from a xenograft culture-free facility [19]. They found six, 13, and none MLV-positive cell lines, respectively. All DNAs were shown to be free of mouse-derived DNA.

The most comprehensive study for XMRV was performed by Hué et al. [15] who screened 411 human cancer cell lines with XMRV primers. They showed that some of the primers used also detected common murine endogenous viral sequences and found nine MLV-positive cell lines. We analyzed 118 of the cell lines also in the current study. Four of them were found positive by Hué et al. but were negative in this study. On the other hand, one cell line (SK-MEL-1) was positive in this investigation and negative in the study of Hué et al. Similar discordances were seen for other cell lines: DG-75 and MEL-JUSO were contaminated as described by Raisch et al. [28] and Deichmann et al. [29], respectively. Both cell lines were not MLV-positive in our study. The discrepancy may be explained by different histories of individual clones of a given cell line. This is also true for the cell line A2780; samples from different laboratories uncovered one MLV-positive cell line which was previously transplanted into a mouse. This demonstrates that cell cultures of a given cell line are not necessarily generally infected or not, but the infection status depends on an individual background.

In the current study, much emphasis was placed on the prevention of false-positive and false-negative results due to PCR inhibition or contamination of DNA or PCR reagents. Hence, false results are unlikely because the PCR products were sequenced and revealed diverse sequences. Additionally, almost all PCR-positive cell lines were also positive in the PERT assay.

The published history of a cell line clone may be a first indication to pinpoint the source of a contamination. As already reported by others [19] and as demonstrated by the different A2780 cell cultures, the passage of tumor cells through mice can be assumed to be a common contamination source. Indeed, a number of cell lines tested positive in the current study were described to have been used in tumorigenicity assays in nude mice. But most cell line descriptions do not explicitly state whether the xenografted cells or the original cell cultures were used afterwards. The contamination can thus be a direct consequence of the tumorigenicity experiment when the xenografted cells were used to establish the cell line, or by contamination of the non-xenografted cells with MLV from mice or infected cell cultures or by cross-contamination of the original cells with xenografted cells. Eight of the 19 contaminated cell cultures were described having been initially or later passaged in mice: KYSE-70 [31], 22RV1 [32], LAMA-87 [33], and DEL [34]. Tumorigenicity assays were also described for the cell lines SK-GT-2 and SCLC-21H [35, 36]. The NCEB-1 cell line is a unique case as this cell line was not used for tumorigenicity assays, but was cultured together with mouse feeder layer during establishment [37]. The cells are described to have acquired several mouse chromosomes [21, 22], which is concordant with our finding that the cell line is positive for MLV as well as for IAP (usually a marker for mouse DNA contaminated human genomic DNA). Whether the cells were contaminated via the transmission of the murine chromosomes including the proviruses or by direct infection with active MLV is unknown. The cells produce active viruses as shown by the PERT assay.

On the other hand, a number of the tested human cell lines are reportedly tumorigenic in mice as determined by xenotransplantation into mice (see datasheets of the DSMZ cell lines), but are negative for MLV. Thus, the handling of mice or murine cells adjacently to the human cell cultures does not necessarily lead to a contamination with murine retroviruses. This situation resembles very much mycoplasma contaminations: the extent of infections seem to depend on the quality of cell culture technique [2]. In many cases, either all or no cell cultures of a given laboratory are contaminated with the same mycoplasma strain. This finding argues for lapses in cell culture technique as contamination source which can also be seen with the high prevalence of false cell lines circulating in many laboratories. Several suggestions had been published to prevent the different types of contamination [38].

We recognized not only a contamination problem regarding XMRV occurrence in the cell lines, but also regarding contamination of the human genomic DNA with mouse-derived DNA detected by IAP-PCR. This problem became also evident in the context of false-positive XMRV contaminations in human prostate carcinoma and chronic fatigue syndrom patients [15, 23]. Performing the appropriate control reactions for the PCR assays as well as examination of the sample DNA for contamination with mouse DNA is recommended. We found that the concurrent extraction of human and mouse DNA can easily lead to cross-contamination of the DNAs. The presence of murine DNA in human genomic DNA preparations can sensitively be detected performing an IAP PCR.

We were able to show by PERT-PCR that most of the contaminated cell cultures also produce MLV. As the viruses use cellular enzymes for the reproduction, it is likely that the cells are affected in many ways. First of all, the viral genome is integrated into the cellular chromosomes. As the integration site is random, the initial infection occurs in many cells at different locations. This increases the chance that an important gene is affected by the integration, resulting in activation or inactivation. Furthermore, the production of new viruses may significantly alter the physiology of the cells because the retroviruses are shed continuously from the cells without lysing and killing the cells. This suggests that the producing cells are constantly altered in their physiology.

It is currently unknown, whether all cells of an MLV positive cell line are infected with the virus or not. If only a subpopulation would carry MLV, the non-infected cells might be separated from the infected cells by single cell cloning. In an effort to move forward in this field, we are currently establishing a fluorescence in-situ hybridization assay to visualize the viral sequences in the cells.

The PERT-PCR assay does not detect MLV reverse transcriptase specifically. Even the use of Mg^2+^- or Mn^2+^-buffers did not clearly differentiate between magnesium and manganesium dependent retroviruses. Thus, the RT activity might also result from other retrovirus contaminations. However, until now only very few reports were published describing the occurrence of retroviruses in human cell lines. For example, the NAMALWA and LCL-HO cell lines are known to be infected with squirrel monkey retroviruses (SMRV) [3]. In case of the MLV-positive NAMALWA sublines, the PERT-PCR signal might completely or in part be generated by SMRV as shown for LCL-HO, which was MLV-negative in the PCR assay, but positive for RT activity. RT activity is also not necessarily an indication for infectious viruses. Although the RT is functional, other defective parts of the viruses might limit the infectivity.

Sequencing of the PCR products and of whole genomes of MLV revealed various genotypes of MLV. All different genotypes could be found in the NCBI databases either as discrete MLV sequence entries or as part of murine genomic DNA sequences. The latter are supposedly proviruses integrated into the murine genome. However, highly related sequences were already found to be contaminants of cell lines: EKVX, LAPC4, VCaP [26], DG-75 [28], RKO [19], the precursor sequences of XMRV, PreXMRV-1 and PreXMRV-2 [32]. Amphotropic murine leukemia viruses are not among the closely related DNA sequences (similarity of only 75%) and can be excluded as contaminants of human and animal cell cultures. Regarding the complete sequences of the MLVs of COLO-677, KYSE-70, and LAMA-87 cell lines highest variations were found in the non-coding regions of the 5´ and 3´ non-coding regions, whereas the coding regions of the gag, pol and env proteins are relatively conserved. The sequence variations in the non-coding regions may have an influence on the infectivity of the viruses produced by the cells. As shown by the infectivity studies, MLV of the COLO-677 cell line was not infectious to the human cell lines MEL-JUSO and NCI-H82. The reason might be the insertion in the U3-region, which is important for the pathogenicity of the viruses [39].

The contamination of human cell cultures with MLV raises the question whether the contaminated cells carry a higher risk regarding the handling of the cell cultures. In contrast to most other viruses, MLV is completely inactivated in the human body by antibody-independent serum components [40–42]. The MLV infectivity had also been assessed with respect to the use of murine retroviruses for gene therapeutic applications. Takeuchi et al. were able to show that in contrast to other C-type retroviruses MLV is not influenced in its sensitivity against human serum components regardless of the production process of the viruses [43]. The sensitivity of the viruses against the complement factors is determined by the virus specific env-gene. Therefore, the pathogenicity of human cell-derived MLV should not be different to murine cell-derived MLV.

In conclusion, we could demonstrate that a) 19 of 577 (3.3%) different human cell lines were contaminated with MLV; b) 17 of the MLV-positive cell lines produce active viruses; c) the infecting MLV constitute at least three evolutionary related groups, d) the retroviruses are transmissible from one infected cell culture to another. Other sources of infection could be heterotransplantation of the human cells and the use of murine feeder layer.

## Supporting Information

S1 Table List of Human Cell Lines Examined. (XLSX)

## References

[1] Dirks WG, Drexler HG. STR DNA typing of human cell lines: detection of intra- and interspecies cross-contamination. Methods Mol Biol. 2013;946: 27–38. 10.1007/978-1-62703-128-8_3 23179824

[2] Uphoff CC, Drexler HG. Mycoplasma contamination of cell cultures In: Flickinger MC, editor. The Encyclopedia of Industrial Biotechnology, Bioprocess, Bioseparation and Cell Technology. 5th ed. New York: Wiley; 2010 pp. 3611–3630.

[3] Uphoff CC, Denkmann SA, Steube KG, Drexler HG. Detection of EBV, HBV, HCV, HIV-1, HTLV-I and-II, and SMRV in human and other primate cell lines. J Biomed Biotechnol. 2010; e904767. 10.1155/2010/904767 PMC2861168 20454443

[4] Kozak CA. The mouse "xenotropic" gammaretroviruses and their XPR1 receptor. Retrovirology. 2010;7: e101. 10.1186/1742-4690-7-101 PMC3009702 21118532

[5] Rein A. Murine leukemia viruses: objects and organisms. Adv Virol. 2011: e403419. 10.1155/2011/403419 PMC3265304 22312342

[6] Miller AD, Wolgamot G. Murine retroviruses use at least six different receptors for entry into Mus dunni cells. J Virol. 1997;71: 4531–4535. 9151846 10.1128/jvi.71.6.4531-4535.1997 PMC191674

[7] Stocking C, Kozak CA. Murine endogenous retroviruses. Cell Mol Life Sci. 2008;65: 3383–3398. 10.1007/s00018-008-8497-0 18818872 PMC4802364

[8] Hempel HA, Burns KH, De Marzo AM, Sfanos KS. Infection of xenotransplanted human cell lines by murine retroviruses: A lesson brought back to light by XMRV. Front Oncol. 2013;3: e156. 10.3389/fonc.2013.00156 PMC3683812 23785669

[9] Kakisi OK, Robinson MJ, Tettmar KI, Tedder RS. The rise and fall of XMRV. Transfus Med. 2013;23: 142–151. 10.1111/tme.12049 23692013

[10] Drexler HG, Dirks W, MacLeod RAF, Quentmeier H, Steube KG, Uphoff CC. DSMZ catalogue of human and animal cell lines. Braunschweig 2015. Available: https://www.dsmz.de/catalogues/catalogue-human-and-animal-cell-lines.html.

[11] Uphoff CC, Drexler HG. Comparative PCR analysis for detection of mycoplasma infections in continuous cell lines. In Vitro Cell Dev Biol Anim. 2002;38: 79–85. 11928999 10.1290/1071-2690(2002)038<0079:CPAFDO>2.0.CO;2

[12] Chang A, Ostrove JM, Bird RE. Development of an improved product enhanced reverse transcriptase assay. J Virol Methods. 1997;65: 45–54. 9128861 10.1016/s0166-0934(96)02168-4

[13] Tamura K, Stecher G, Peterson D, Filipski A, Kumar S. MEGA6: Molecular Evolutionary Genetics Analysis version 6.0. Mol Biol Evol. 2013;30: 2725–2729. 10.1093/molbev/mst197 24132122 PMC3840312

[14] Hohn O, Krause H, Barbarotto P, Niederstadt L, Beimforde N, Denner J, et al Lack of evidence for xenotropic murine leukemia virus-related virus (XMRV) in German prostate cancer patients. Retrovirology. 2009;6: e92. 10.1186/1742-4690-6-92 PMC2770519 19835577

[15] Hue S, Gray ER, Gall A, Katzourakis A, Tan CP, Houldcroft CJ, et al Disease-associated XMRV sequences are consistent with laboratory contamination. Retrovirology. 2010;7: e111. 10.1186/1742-4690-7-111 PMC3018392 21171979

[16] Cmarik JL, Troxler JA, Hanson CA, Zhang X, Ruscetti SK. The human lung adenocarcinoma cell line EKVX produces an infectious xenotropic murine leukemia virus. Viruses. 2011;3: 2442–2461. 10.3390/v3122442 22355448 PMC3280514

[17] Lee YJ, Jeong BH, Choi EK, Carp RI, Kim YS. Complete genome sequences of new xenotropic murine leukemia viruses from the senescence-accelerated mouse (SAM): molecular and phylogenetic analyses. PLOS ONE. 2013;8: e55669 10.1371/journal.pone.0055669 23393596 PMC3564811

[18] Malmsten A, Ekstrand DH, Akerblom L, Gronowitz JS, Kallander CF, Bendinelli M, et al A colorimetric reverse transcriptase assay optimized for Moloney murine leukemia virus, and its use for characterization of reverse transcriptases of unknown identity. J Virol Methods. 1998;75: 9–20. 9820570 10.1016/s0166-0934(98)00091-3

[19] Zhang YA, Maitra A, Hsieh JT, Rudin CM, Peacock CD, Karikari C, et al Frequent detection of infectious xenotropic murine leukemia virus (XMLV) in human cultures established from mouse xenografts. Cancer Biol Ther. 2011;12: 617–628. 21750403 10.4161/cbt.12.7.15955 PMC3218386

[20] Kuff EL, Lueders KK. The intracisternal A-particle gene family: structure and functional aspects. Adv Cancer Res. 1988;51: 183–276. 3146900 10.1016/s0065-230x(08)60223-7

[21] Camps J, Salaverria I, Garcia MJ, Prat E, Bea S, Pole JC, et al Genomic imbalances and patterns of karyotypic variability in mantle-cell lymphoma cell lines. Leuk Res. 2006;30: 923–934. 16448697 10.1016/j.leukres.2005.11.013

[22] Drexler HG, MacLeod RA. Mantle cell lymphoma-derived cell lines: unique research tools. Leuk Res. 2006;30: 911–913. 16563503 10.1016/j.leukres.2006.02.015

[23] Oakes B, Tai AK, Cingoz O, Henefield MH, Levine S, Coffin JM, et al Contamination of human DNA samples with mouse DNA can lead to false detection of XMRV-like sequences. Retrovirology. 2010;7: e109. 10.1186/1742-4690-7-109 PMC3022687 21171973

[24] Zheng H, Jia H, Shankar A, Heneine W, Switzer WM. Detection of murine leukemia virus or mouse DNA in commercial RT-PCR reagents and human DNAs. PLOS ONE. 2011;6: e29050 10.1371/journal.pone.0029050 22205995 PMC3243700

[25] McAllister RM, Nicolson M, Gardner MB, Rongey RW, Rasheed S, Sarma PS, et al C-type virus released from cultured human rhabdomyosarcoma cells. Nat New Biol. 1972;235: 3–6. 4111969 10.1038/newbio235003a0

[26] Sfanos KS, Aloia AL, Hicks JL, Esopi DM, Steranka JP, Shao W, et al Identification of replication competent murine gammaretroviruses in commonly used prostate cancer cell lines. PLOS ONE. 2011;6: e20874 10.1371/journal.pone.0020874 21698104 PMC3117837

[27] Stang A, Petrasch-Parwez E, Brandt S, Dermietzel R, Meyer HE, Stuhler K, et al Unintended spread of a biosafety level 2 recombinant retrovirus. Retrovirology. 2009;6: e86. 10.1186/1742-4690-6-86 PMC2760500 19772602

[28] Raisch KP, Pizzato M, Sun HY, Takeuchi Y, Cashdollar LW, Grossberg SE. Molecular cloning, complete sequence, and biological characterization of a xenotropic murine leukemia virus constitutively released from the human B-lymphoblastoid cell line DG-75. Virology. 2003;308: 83–91. 12706092 10.1016/s0042-6822(02)00074-0

[29] Deichmann M, Huder JB, Kleist C, Naher H, Schupbach J, Boni J. Detection of reverse transcriptase activity in human melanoma cell lines and identification of a murine leukemia virus contaminant. Arch Dermatol Res. 2005;296: 345–352. 15630577 10.1007/s00403-004-0501-4

[30] Takeuchi Y, McClure MO, Pizzato M. Identification of gammaretroviruses constitutively released from cell lines used for human immunodeficiency virus research. J Virol. 2008;82: 12585–12588. 10.1128/JVI.01726-08 18842727 PMC2593302

[31] Shimada Y, Imamura M, Wagata T, Yamaguchi N, Tobe T. Characterization of 21 newly established esophageal cancer cell lines. Cancer. 1992;69: 277–284. 1728357 10.1002/1097-0142(19920115)69:2<277::aid-cncr2820690202>3.0.co;2-c

[32] Paprotka T, Delviks-Frankenberry KA, Cingoz O, Martinez A, Kung HJ, Tepper CG, et al Recombinant origin of the retrovirus XMRV. Science. 2011;333: 97–101. 10.1126/science.1205292 21628392 PMC3278917

[33] Champelovier P, Valiron O, Michele J, Dominique L, Seigneurin D. Selection and characterization of an erythroeosinophilic subclone (LAMA-87) and an eosinophilic subclone (LAMA-88) from the multipotential cell line LAMA-84. Leuk Res. 1994;18: 903–918. 7996872 10.1016/0145-2126(94)90102-3

[34] Barbey S, Gogusev J, Mouly H, Le Pelletier O, Smith W, Richard S, et al DEL cell line: a "malignant histiocytosis" CD30+ t(5;6)(q35;p21) cell line. Int J Cancer. 1990;45: 546–553. 2307542 10.1002/ijc.2910450329

[35] Altorki N, Schwartz GK, Blundell M, Davis BM, Kelsen DP, Albino AP. Characterization of cell lines established from human gastric-esophageal adenocarcinomas. Biologic phenotype and invasion potential. Cancer. 1993;72: 649–657. 8334620 10.1002/1097-0142(19930801)72:3<649::aid-cncr2820720305>3.0.co;2-l

[36] Bepler G, Jaques G, Neumann K, Aumuller G, Gropp C, Havemann K. Establishment, growth properties, and morphological characteristics of permanent human small cell lung cancer cell lines. J Cancer Res Clin Oncol. 1987;113: 31–40. 3029138 10.1007/BF00389964 PMC12248341

[37] Saltman DL, Cachia PG, Dewar AE, Ross FM, Krajewski AS, Ludlam C, et al Characterization of a new non-Hodgkin's lymphoma cell line (NCEB-1) with a chromosomal (11:14) translocation [t(11:14)(q13;q32)]. Blood. 1988;72: 2026–2030. 2848599

[38] Uphoff CC, Drexler HG. Prevention of mycoplasma contamination in leukemia-lymphoma cell lines. Human Cell. 2001;14: 244–247. 11774744

[39] Rabson AB, Graves BJ. Synthesis and procession of viral RNA In: Coffin JM, Hughes SH, Varmus HE, editors. Retroviruses. Cold Spring Harbor (NY): Cold Spring Harbor Laboratories Press; 1997 pp. 205–262. 21433339

[40] Welsh RM Jr., Cooper NR, Jensen FC, Oldstone MB. Human serum lyses RNA tumour viruses. Nature. 1975;257: 612–614. 170540 10.1038/257612a0

[41] Welsh RM. Host cell modification of lymphocytic choriomeningitis virus and Newcastle disease virus altering viral inactivation by human complement. J Immunol. 1977;118: 348–354. 830758

[42] Sherwin SA, Benveniste RE, Todaro GJ. Complement-mediated lysis of type-C virus: effect of primate and human sera on various retroviruses. Int J Cancer. 1978;21: 6–11. 75191 10.1002/ijc.2910210103

[43] Takeuchi Y, Cosset FL, Lachmann PJ, Okada H, Weiss RA, Collins MK. Type C retrovirus inactivation by human complement is determined by both the viral genome and the producer cell. J Virol. 1994;68: 8001–8007. 7966590 10.1128/jvi.68.12.8001-8007.1994 PMC237263

